# Assessment of Anticancer and Antimicrobial Potential of Bioactive Metabolites and Optimization of Culture Conditions of *Pseudomonas aurantiaca* PB-St2 for High Yields

**DOI:** 10.4014/jmb.2311.11041

**Published:** 2025-02-14

**Authors:** Mahnoor Zameer, Izzah Shahid, Rahman Shahzaib Saleem, Deeba Noreen Baig, Maryam Zareen, Kauser Abdulla Malik, Samina Mehnaz

**Affiliations:** 1 Kauser Abdulla Malik School of Life Sciences, Forman Christian College (A Chartered University), Ferozepur Road, Lahore 54600, Pakistan; 2 Institute of Multidisciplinary Research in Applied Biology, Public University of Navarra, Campus Arrosadia, Pamplona 31006, Spain; 3 Department of Chemistry and Chemical Engineering, Syed Babar Ali School of Science and Engineering, Lahore University of Management Sciences, Lahore 54792, Pakistan

**Keywords:** Anticancer, antimicrobial, bioactive metabolites, chromatography, *P. aurantiaca*

## Abstract

The following study aimed to characterize the biological potential of the purified compounds of *Pseudomonas aurantiaca* PB-St2. Optimization of temperature and incubation time of 32°C and 72 h yielded the highest crude extract weight and optical density of bacterial culture. HPLC analysis of the crude metabolite extract (purified using gravitational column chromatography) showed three fractions named as PC1, PC2, and PC3. HPLC-purified fractions were subjected to LC-MS/MS analysis and the data was compared using reference library. Fraction PC1 was identified as mupirocin, PC2 as phenazine-1-carboxylic acid (PCA), and PC3 as the mixture of three compounds including pyoluteorin, PCA and 2-hydroxyphenazine (2-OH-phz). Fungicidal potential of the purified compounds was assessed against phytopathogens including *Fusarium equiseti*, *Fusarium incarnatum*, *Alternaria alternata*, and *Colletotrichum falcatum*. Fraction PC3 showed the highest fungicidal activity of ~89%, whereas, the least antifungal activity (~27%) was noted for mupirocin. Antibacterial activity of the purified compounds against Gram-positive pathogen *Bacillus cereus*, and Gram-negative pathogens *Pseudomonas aeruginosa*, *Salmonella enterica*, and *Klebsiella oxytoca* was also assessed. Fraction PC3 demonstrated the highest antibacterial activity against *B. cereus* and *P. aeruginosa* showing 1.8 cm, and 0.9 cm zones of inhibition, respectively. Against *K. oxytoca* and *S. enterica*, the antibacterial activity of PB-St2 crude extract was slightly higher than the fraction PC3. The fraction PC3 also demonstrated the highest IC_50_ against HepG-2 and SF767 cancer cell lines at 25 μg and 20 μg concentrations, respectively. The multifaceted attributes of *P. aurantiaca* PB-St2 make it an ideal candidate for agricultural and pharmacological applications.

## Introduction

*Pseudomonas* spp. have gained enormous attention due to production of a wide range of secondary metabolites. Several species of fluorescent pseudomonads including *P. fluorescens*, *P. aureofaciens*, *P. putida*, *P. chlororaphis* and *P. aurantiaca* have been reported for plethora of secondary metabolites with diverse biological applications. Among these, the most diverse are *P. aurantiaca* strains which produce varied classes of metabolites comprising phenazines, pyrrolnitrin, pyoluteorin, diketopiperazines, and cyclic-peptides [1-2]. Phenazines are regarded as the most dominant class of *P. aurantiaca* and *P. chlororaphis* metabolites with several derivatives and enormous biological potential. The most abundant of these phenazines are phenazine-1-carboxylic acid (PCA), 2-hydroxy-phenazine-1-carboxylic acid (2-OH-PCA), 2-hydroxy-phenazine (2-OH-phz), phenazine carboxamide (PCN), and pyocyanin which is termed as the blue phenazine [4].

Phenazine-1-carboxylic acid (PCA) is a reported biofungicide and has demonstrated biocontrol of several notorious fungal phytopathogens. Commercially available fungicide Shenqinmycin is isolated and extracted from *Pseudomonas* sp. and is effective against *Fusarium* spp., *Pythium* spp., and *Gaeumannomyces graminis* var. *tritici* [5, 6]. Minimal toxicity of PCA towards humans and animals in comparison to commercially available chemical fungicides and eco-friendliness adds to its superiority and demand [5]. In addition to PCA, another widely studied phenazine is 2-hydroxy-phenazine (2-OH-phz) with demonstrated antimicrobial potential against *Rhizoctonia solani*, *Phytophthora parasitica* var. *nicotianae*, *Diaporthe citri*, *Klebsiella oxytoca*, *Shigella castellani*, and *Salmonella* sp. [7, 8]. These phenazines compounds have been subjected to rigorous derivatization to assess the increase or decrease of their biological potential. Moreover, several chemically synthesized phenazine derivatives have been documented for anticancer properties, nonetheless, chemical synthesis of phenazines is costly, eco-damaging, and results in lower yields. Hence, the microbial synthesis of phenazines offer an attractive alternative and requires a comprehensive profiling of their biological activities for better exploitation and use.

Equally contributing to the biological potential of *Pseudomonas* spp. are the other metabolites such as pyrrolnitrin, pyoluteorin, mupirocin, and cyclic-peptides. Mupirocin is a characteristic antibiotic produced by pseudomonads and is widely used for the treatment of primary and secondary skin infections [9]. However, emerging bacterial resistance against mupirocin and its decreasing efficacy to curtail infections is impulsive to explore new bacterial strains capable of mupirocin biosynthesis. Similarly, pyoluteorin has been majorly reported from *Pseudomonas protegens* strain Pf-5 and showcases antibacterial activity against phytopathogen of maize *Pantoea ananatis* [10]. However, less is known for its antibacterial potential against mammalian pathogens and optimal condition for the maximum yields of this compound. *Pseudomonas aurantiaca* PB-St2 strain used in this study was analyzed for the optimal production of phenazines and other metabolites following different parameters. Understanding of bacterial kinetics and physicochemical properties is necessary as niche plays a pivotal role in the growth and sustenance of an organism. Parameters such as temperature, pH, electrical conductivity, density, viscosity, and chemical composition influence the bacterial growth, metabolites production, and enzymatic activities, ultimately contributing to the biological potential of a particular organisms [11]. Besides, these parameters need to be optimized to take full advantage of the beneficial compounds with enormous biological activities produced by these microbes. Besides, a complete profiling of these metabolites will allow to comprehend their multifold uses in pharmaceutical, agricultural, and clinical settings. Metabolites of *P. aurantiaca* strain PB-St2 characterized in this study were subjected to detailed profiling to assess their biological activities. The results obtained are promising and prove the candidature of the characterized compounds for their multifaceted use in pharmaceutical and agricultural applications.

## Materials and Methods

### Bacterial Strain *Pseudomonas chlororaphis* subsp. *aurantiaca* PB-St2

A pre-characterized strain of *Pseudomonas chlororaphis* subsp. *aurantiaca* PB-St2 was used for this study. The strain has been already characterized for its secondary metabolites production and its genome sequence is deposited at DDBJ/ EMBL/GenBank under the accession number AYUD00000000 [12]. PB-St2 was revived from glycerol stock in Kings’ B medium [g/l= Peptone: 20, MgSO_4_·7H_2_O: 1.5, K_2_HPO_4_: 1.5, Glycerol: 20 ml] and incubated at 150 rpm and 28°C for 48 h [13]. The culture was assessed for purity and used for subsequent experiments. Details of all the strains used in this study have been provided in Table 1.

### Optimization of Parameters for Higher Yield of PB-St2 Secondary Metabolites

Temperature and incubation period were optimized for enhanced yield of PB-St2 secondary metabolites. Initially, PB-St2 was grown in 100 ml Kings’ B broth at seven different temperatures including 28°C, 30°C, 32°C, 34°C, 36°C, 38°C, and 40°C for 24 h and the results were recorded. Based on these results, temperature was kept constant and PB-St2 was cultivated at four different incubation periods comprising 24 h, 48 h, 72 h, and 96 h. Samples were harvested following extraction of metabolites using a previously reported method [13]. Extracts showing higher secondary metabolites yield were subjected to HPLC analysis [HPLC system with 2998 photodiode-array (PDA) detector (Waters, USA)]. Samples were injected onto Nucleosil C18 column [4.6 × 250 mm, 5 μm; Macherey-Nagel, Germany] and the resultant peaks were analyzed for the relative production and quantification of phenazines. To minimize error in quantification, samples were analyzed in triplicates.

For characterization of phenazine-1-carboxylic acid (PCA), standard stock solution of PCA (Sigma Aldrich, Merck, Germany) was prepared by dissolving 1 mg PCA in 1 ml absolute methanol (Merck). Further dilutions were made from the stock solution to formulate a standard curve following HPLC analysis. Each concentration was dissolved in methanol to make the volume up to 1 ml. The selected concentrations were 10, 20, 30, 40, 50, 60, 70, 80, 90 and 100 ppm. For HPLC analysis, 10 μl injection volume was used and a gradient of two solvent systems was used to separate the metabolites (Table 2). Total HPLC runtime was 30 min, and the flow rate was 1 ml/min. Sample extracts were individually dissolved in 1 ml methanol, filtered (PVDF sterile syringe filters of 0.2 μm; Filter-bio, China), and used for HPLC analysis. Peak area and retention time were used for the quantification of PCA from each sample. Chromatograms were analyzed using HPLC software (Empower^®^3) for absolute quantification in parts per million (ppm).

A Response surface methodology (RSM; Design-Expert, Stat-Ease 360^®^) based on Box Behnken Design (BBD) was implemented to study the correlation of two variables, *i.e.*, temperature and incubation period, with the crude extract weight, optical density, and PCA production (response) of *P. aurantiaca* PB-St2 (Table 3). A total of thirteen experimental runs were conducted in duplicates to optimize the response of the variables (Table S1). Analysis of variance (ANOVA) for Quadratic Model was used with the significance level *p*-value (*p* ≤ 0.05), F-value and the coefficient R^2^ to define the significance of each factor. Three-dimensional surface plots were made to graphically represent the maximum yield under different incubation periods and temperature range [14].

Temperature and incubation period showing the highest values for crude extract weight and PCA quantification, were selected for bulk production and extraction of phenazines.

### Isolation and Purification of Phenazines

**Bulk Extraction**

A single colony of *P. aurantiaca* PB-St2 was transferred to 10 ml King’s B broth. The culture was incubated overnight and 50 ml of this culture (1%v/v of the primary inoculum) was transferred to 5 L King’s B broth. The culture was placed in shaking incubator at 32°C and 150 rpm for 96 h. Following incubation, the culture was centrifuged at 3,600 rpm (HERMLE Z 513K Centrifuge, Germany) for 40 min. Supernatant was acidified to pH 2 with 1 N HCl and extracted twice with equal volume of ethyl acetate (Merck). The organic layer was collected, dehydrated with anhydrous sodium sulphate (Honeywell, Germany), and evaporated to dryness via rotary evaporator (Witeg, HS-2005S-N, Germany) at 40°C. Residual extract was re-suspended in minimum amount of chloroform (Merck) and used for thin layer chromatography analysis.

### Thin Layer Chromatography (TLC)

A number of solvent systems were compared for the analysis of chloroform soluble fraction of PB-St2 extract using thin layer chromatography (TLC Silica Gel 60 F_254_, 20 × 20 cm, Merck) analysis. Glass capillary tube was used to spot the crude extract on the TLC sheets. The sheets were suspended in the mobile phase and were observed after mobile phase travelled to 3/4^th^ of the total sheet area. The sheets were air-dried and visualized with 254 nm UV light (Gel Documentation system, France). Retardation factor (*Rf*) value for each separated spot was calculated using the standard formula [15]. The best mobile phase for TLC which significantly separated the compounds from each other was selected for column chromatography.

### Column Chromatography

The glass column used was 35 cm long and 3.5 cm wide. The column was thoroughly washed with detergent and then with 70% diluted ethanol. For column packing, 10-15 g silica gel 60 (0.063-0.200 mm, Merck) was suspended in *n*-hexane (Merck) to make the silica bed of 6 inches. The chloroform extract was re-suspended in 20-30 ml chloroform and sonicated for 1-2 min to completely dissolve any suspended particles. Subsequently, 3 to 4 g of silica were added in the sample and dried using rotary evaporator to get a powdery product as dry load of the column [16].

Gradient solvent system of *n*-hexane:ethyl acetate (1:0, 9:1,8:2, 7:3, 6:4, 1:1, 4:6, 3:7, 2:8, 1:9, 0:1) was used for elution. Fractions collected from the column were spotted on TLC plate and observed under UV light (254 nm). Colored spots depicted the elution of compounds and approximately 1-2 ml of the eluate was collected in each tube. Similar fractions were mixed based on TLC analysis. Larger fractions were concentrated using rotary evaporator, while the ones with lower amount were allowed to concentrate at room temperature for 24-48 h. All concentrated fractions were weighed and 5 ml of absolute ethanol were added to each remaining concentrate. Ethanol was allowed to evaporate at room temperature following the crystallization of compounds. Resultant crystalized compounds were dissolved in 5-10 ml absolute ethanol and left at room temperature for 24 h. Later, the crystals were filtered via filter paper by passing more ethanol through the crystals 5-6 times (crystal washing). After thorough washing, the crystals were preserved at room temperature.

### Purification of Compounds through HPLC

The purity of the collected crystals was checked by HPLC. For analysis, 1 mg of the crystals was dissolved in 50 μl of DMSO (Honeywell). Methanol was added to make the volume up to 1 ml and 25-μl sample was used for HPLC analysis following the solvent gradient mentioned in Table 2. Fraction collector connected with HPLC was used to collect purified fractions. Each collected fraction was concentrated by using rotary evaporator.

### Liquid Chromatography-Mass Spectrometry (LC-MS/MS) Analysis

HPLC-purified fractions were subjected to LC-MS/MS analysis. Samples were injected onto mass spectrometer (Infinity Prime II, 6470, Agilent, USA) using Eclipse Plus C18 ZORBAX column (3.0 × 50 mm, 1.8 μm, Agilent). The mobile phase used was: (A) water with 0.04% acetic acid, (B) 100% acetonitrile with 0.4 ml/min flow rate, and 40 min run time. Scans with signals corresponding m/z [M+H]^+^ mode were matched with the reference library and the chromatograms of compounds previously isolated and characterized from PB-St2 [13, 17].

### Bioactivities of Purified Compounds of PB-St2

Purified compounds of *P. aurantiaca* PB-St2 were assessed for antimicrobial, antifungal, and anticancer potential. Stocks were prepared from individual compounds by dissolving 1 mg of each HPLC-purified compound in DMSO (200 μl) and methanol (800 μl) to make the stock volume of 1 mg/ml. Negative control (DMSO: methanol; 200 μl:800 μl/ml) was also used. These stocks were used for antifungal, antibacterial and anticancer assays.

### Antifungal Activity

*Fusarium equiseti*, *F. incarnatum*, *Colletotrichum falcatum*, *Alternaria alternata* 1, and *Alternaria alternata* 2 were used for antifungal screening. Fungal phytopathogens were grown for 4-5 days, on potato dextrose agar (PDA; bio world, USA) at 25°C. Sterilized filter paper discs (9 mm in diameter) individually loaded with 100 μg of each compound, were placed 2 cm away from the fresh fungal plug (1 mm^2^) and incubated for 5-6 days at 25°C. Percentage inhibition of fungal growth was measured according to the standard formula [18, 19]. Hyphal de-morphogenesis was observed under Stereo microscope at 40X and fluorescent microscope at 254 nm, 40X (Olympus ix83).

### Antibacterial Activity

The antibacterial activity of HPLC-purified compounds against bacterial pathogens including *Bacillus cereus*, *P. aeruginosa*, *S. enterica*, and *Klebsiella oxytoca* was checked by using agar disc-diffusion method [20]. All pathogens were cultivated for overnight growth in 10 ml LB broth at 37°C. A 20 ml aliquot of each bacterial culture was individually spread on LB agar plates. Sterilized filter paper discs (6 mm in diameter) were separately coated with 25, 50, 75, 100 μg concentrations of each compound and placed on the LB plates. Positive (PB-St2 crude extract) and negative controls (200 μl DMSO + 800 μl methanol) were also tested. All the plates were prepared in triplicates and incubated for 24 h at 37°C. Antibacterial activity was recorded by measuring the diameter of the inhibition zones.

### Anticancer Activity

The HPLC-purified compounds were tested for their cell toxicity potential on human liver cancer cell line (HepG-2), and tumor cell line of glial cells of central nervous system (SF767). Protocol of MTT (3-(4,5-Dimethylthiazol-2-yl)-2,5-Diphenyltetrazolium Bromide) assay was followed as depicted by Van Meerloo and coworkers [21]. Initially, 5,000 cells of each cell line were seeded in 96-well microtiter plate (SPL, Republic of Korea) using Dulbecco’s Modified Eagle Medium (DMEM; Gibco, UK) and incubated at 37°C (5% CO_2_ level) for 24 h. Following this, each cell line was cultured separately with varying concentrations (2.5, 5, 10, 15, 20, 25, 50, 75, 125 and 250 μg) of the HPLC-purified compounds and re-incubated at 37°C and 5% CO_2_ level for next 24 h. After incubation, medium from each well was completely removed, and 200 μl of fresh medium and 20 μl of MTT solution (5 mg/ml solution in PBS; Sigma-Aldrich) were added in each well following incubation at 37°C and 5%CO_2_ level for 2 h [22]. Next, 200 μl of solubilizing solution (40% DMSO, 2% glacial acetic acid, 16% sodium dodecyl sulfate; pH 4.7) was added to each well and incubated for additional 2 h at 37°C. Experiment was done in duplicate and results were recorded using ELISA reader (Bio-Rad, I-Mark, USA) at 595 nm wavelength.

## Results

### Optimization of Parameters for Higher Yield of *P. aurantiaca* PB-St2 Metabolites

**Fig. 1:**
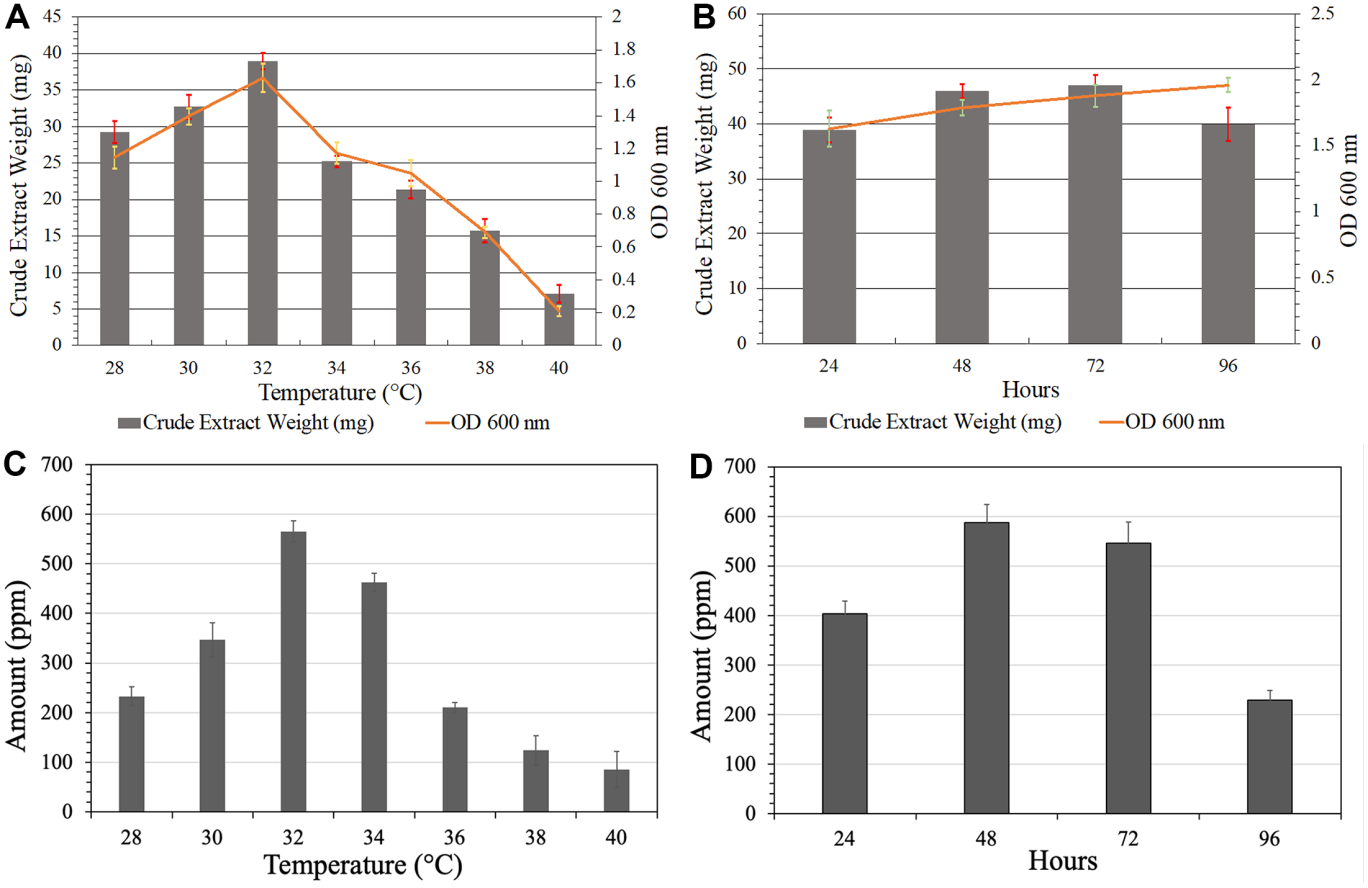
Graphical representation of optimization of high yields of crude extract weight, optical density and PCA production of PB-St2. (**A**) measurement of cell density and crude extract weight at different temperatures, (**B**) measurement of cell density and crude extract weight at different incubation times, (**C**) average quantification of PCA at different temperature ranges, (**D**) average quantification of PCA at different incubation times. The error bars represent standard deviation and results are the means of three replicates.

The highest optical density (OD) value of 1.63 of PB-St2 culture was observed when cultivated at 32°C for 24 h. Culture density increased upon raising the temperature from 28-32°C for 1.14-1.63 OD. Decline in the cell number was observed when temperature was increased above 32°C, and the lowest OD was observed at 40°C (0.21). Average values of OD observed for 34, 36 and 38°C were 1.17, 1.05, and 0.69, respectively. A similar pattern was observed for crude extract weight where the maximum weight was recorded at 32°C (38.95 mg/100 ml) and the lowest at 40°C (9.6 mg/100 ml). Average values for 28, 30, 34, 36 and 38°C were 30.05, 34, 29.5, 23.2, and 17.85 mg/100 ml, respectively (Fig. 1A). At 32°C, maximum OD and the highest extract weight were recorded, therefore, this temperature was further used to optimize the incubation period. After 96 h of incubation, maximum optical density (OD) of 1.96, followed by 72 h (1.88), 48 h (1.79) and 24 h (1.63) h, respectively, was observed. The highest crude extract weight from the culture was recorded after 72 h (47 mg), followed by 48 h (45.93 mg), 96 h (39.9 mg), and 24 h (38.95 mg) (Fig. 1B).

HPLC quantification revealed that the maximum PCA production was at 32°C (564.826 ppm) followed by 34°C (462.627 ppm). The lowest production of PCA was observed at 40°C (85.3 ppm) (Fig. 1C). The 48 h of incubation produced maximum amount of PCA (587.874 ppm) followed by 72 h (545.7755 ppm). The lowest production of PCA was recorded after 96 h (228.777 ppm) (Fig. 1D). Standard curve was generated by using various concentrations of PCA standard (Fig. S1).

**Fig. 2:**
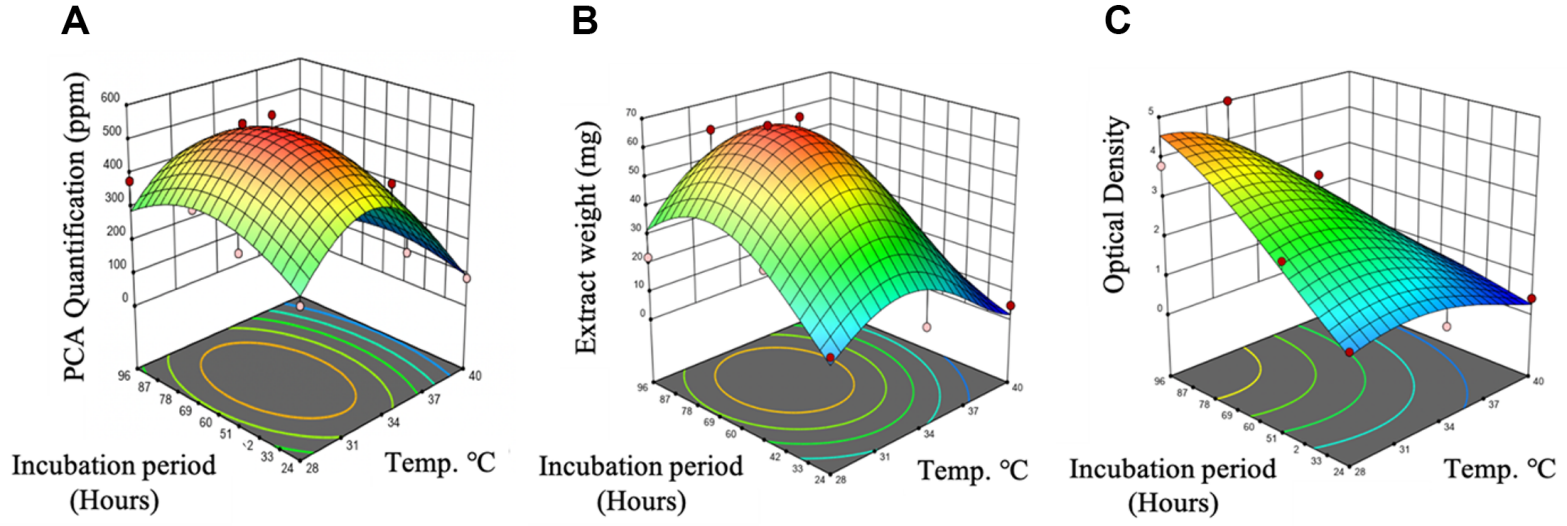
Three dimensional (3D) surface plots based on experimental data showing the effect of temperature and incubation period on PCA quantification (A) extract weight (B) and optical density (C). Results are the means of two replicates.

According to the experimental BBD design, a total of thirteen experimental runs were carried out in duplicates. Data for both variables (A, B) was significant for response processes when analyzed under RSM. The *p*-values of factor A and B were less than 0.05, which indicated that the variables had significant effect on the PCA production, extract weight and optical density of PB-St2. In terms of PCA quantification, *p*-value of A (0.0098) was significant indicating that temperature affected the production of PCA as compared to the incubation period (*p*-value: 0.9860). For extract weight, *p*-value of both variables A (0.0211) and B (0.0211) indicated its influence on the extract weight of PB-St2. Similarly, *p*-value of A (0.0004) and B (0.0007) being highly significant implied the influence of the variables on the optical density (Fig. S2). Fig. 2 illustrates 3-D graphs showing the effect of incubation period and temperature on PCA quantification, extract weight, and optical density. The data inferred that temperature affected the concentration of PCA more than the incubation period (Fig. 2A). Moreover, both temperature and incubation period impacted the extract weight and optical density of PB-St2 (Fig. 2B and 2C).

### Isolation and Purification of Phenazines

**Extraction and Thin Layer Chromatography**

**Fig. 3:**
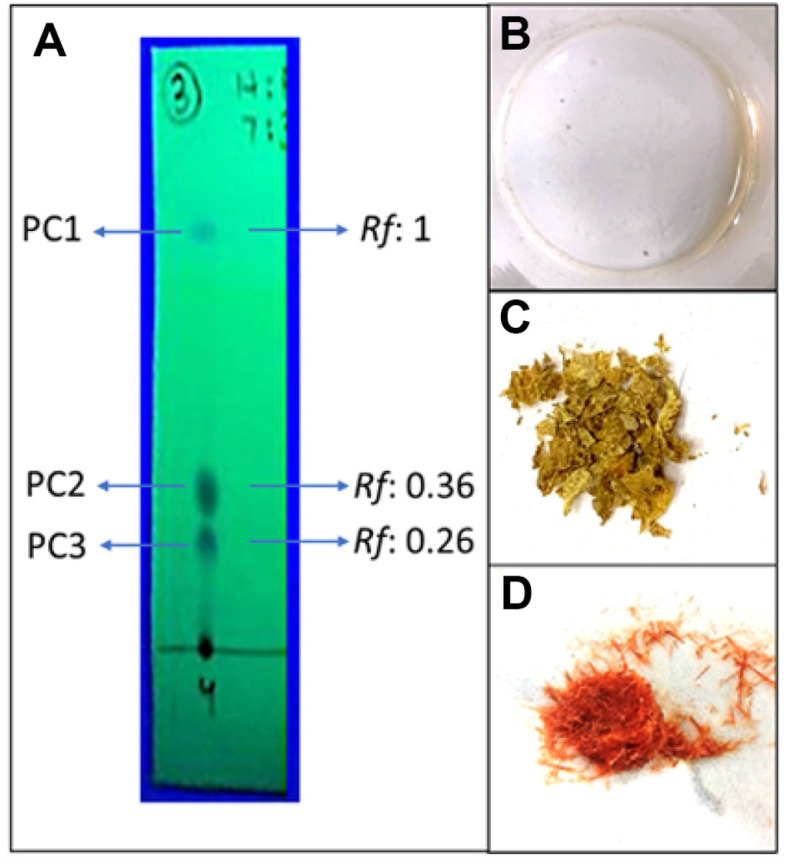
Extraction and isolation of compounds from *P. aurantiaca* PB-St2. Thin layer chromatography (TLC) of PB-St2 crude extract with mobile phase ethyl acetate: n-hexane (7:3) showing three compounds PC1, PC2 and PC3 (**A**) concentrated fractions revealing PC1 as a greasy textured compound (**B**) yellow-color crystals of PC2 (**C**) and dark orange colored crystals of PC3 (**D**).

The crude extract (4.93 g) was subjected to multiple solvent systems for separation of metabolites. The best one amongst them was a combination of non-polar (*n*-hexane) and polar (ethyl acetate) solvents. A gradient of solvent system with the combination of *n*-hexane:ethyl acetate was used for TLC (Fig. S3). TLC with 100% *n*-hexane as solvent system did not show the separation of any compound. Gradual increase in polar solvent, ethyl acetate, revealed the presence of three compounds. The ratio 7:3 (*n*-hexane:ethyl acetate) revealed three spots at different *Rf* values under 254 nm. The topmost spot (PC1) was at *Rf* value 1, the middle one (PC2) at *Rf* 0.36, and the bottom one (PC3) was at *Rf* 0.26 (Fig. 3A).

### Column Chromatography

Gradient of *n*-hexane:ethyl acetate (1:0, 9:1, 8:2, 7:3, 6:4, 5:5, 4:6, 3:7, 2:8, 1:9, 0:1) was run on column. Table S2 summarizes the complete procedure of gravitational column chromatography depicting ratios of the solvents used, number of fractions collected containing the compounds, amount of mobile phase used for each ratio, *Rf* values of the collected compounds, and yield of each collected compound. Fractions were collected after spots appeared on the TLC sheets, visualized under 254 nm. Fraction containing PC1 eluted first at the ratio 7:3 (*n*-hexane: ethyl acetate). Eight fractions (F1-F8; each of 2 ml volume) of the eluate, contained PC1. All fractions were concentrated and 19.7 mg of an off-white, greasy textured compound was obtained (Fig. 3B). The fraction containing PC2 eluted at the ratio 4.5:5.5 (*n*-hexane: ethyl acetate). The PC2 containing fractions (F15-F28), after concentration, showed shiny yellow-color crystals weighing 70.35 mg (Fig. 3C). The last fraction containing PC3, eluted under the ratio 4.4:5.6 (*n*-hexane: ethyl acetate). Fractions containing PC3 (F31-F48) appeared dark yellow in color, however, concentrated and dried fractions revealed orange-red color crystals weighing 120 mg (Fig. 3D). TLC of all the collected fractions was carried out to check the purity and observed under UV 254 nm.

### HPLC Analysis and Fraction Collection

The above collected fractions containing different compounds were run on HPLC to check their purity. The fractions containing PC1 and PC2 showed single peaks, whereas the third fraction containing PC3 revealed three sub-peaks with close retention time (RT) values and each sub-peak was further collected separately by using a fraction collector. The collected sub-peaks were termed as P1, P2, and P3 (Fig. S4). Fresh eluates were concentrated and preserved at -20°C until further use.

### LC-MS/MS Analysis

**Fig. 4:**
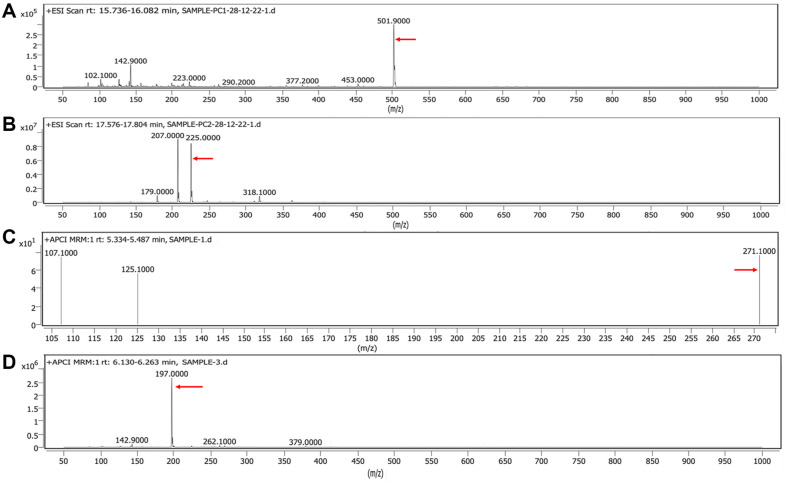
LC-MS chromatograms of purified compounds. PC1 as mupirocin m/z [M+H]^+^ 501.9 (**A**), PC2 and P2 as phenazine-carboxylic acid m/z [M+H]^+^ 225.0 (**B**), P1 as pyoluteorin m/z [M+H]^+^ 271.1 (**C**), and P3 as 2-hydroxyphenazine (2- OH-phz) m/z [M+H]^+^ 197.0 (**D**).

Compounds were identified by searching MS/MS library and standard reference data of compounds pre-identified from PB-St2. PC1 was identified as mupirocin (Mu) m/z [M+H]^+^ 501.9. Compound PC2 showed homology with phenazine-1-carboxylic acid (PCA) m/z [M+H]^+^ 225 while PC3 showed three different compounds in it. In PC3, P1 was identified as pyoluteorin (plt) m/z [M+H]^+^ 271, P2 matched with phenazine-1-carboxylic acid (PCA), and P3 was identified as 2-hydroxyphenazine (2-OH-phz) m/z [M+H]^+^ 197.0 (Fig. 4). MS/MS chromatograms of these compounds are given in Figs. S5-S8.

### Bioactivities of Purified Compounds of PB-St2

**Antifungal Activity**

**Fig. 5:**
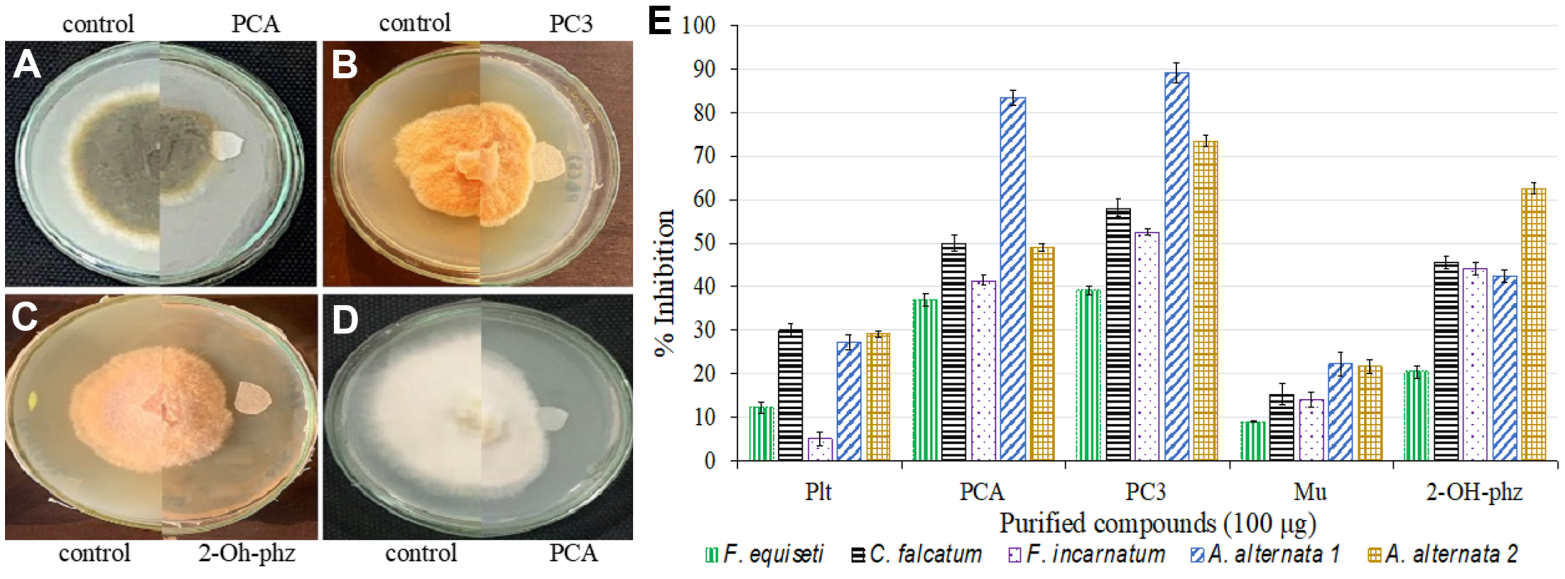
Antifungal plate assay of *P. aurantiaca* compounds showing antagonistic activity of PCA against *Alternaria alternata*1 (A) PC3 against *Fusarium equiseti* (B) 2-OH-phz against *Colletotrichum falcatum* (C) and PCA against *Fusarium incarnatum* (D). Graph representing average percent inhibition of compounds against selected fungal phytopathogens (E). The error bars represent standard deviation where results are the means of two replicates.

**Fig. 6:**
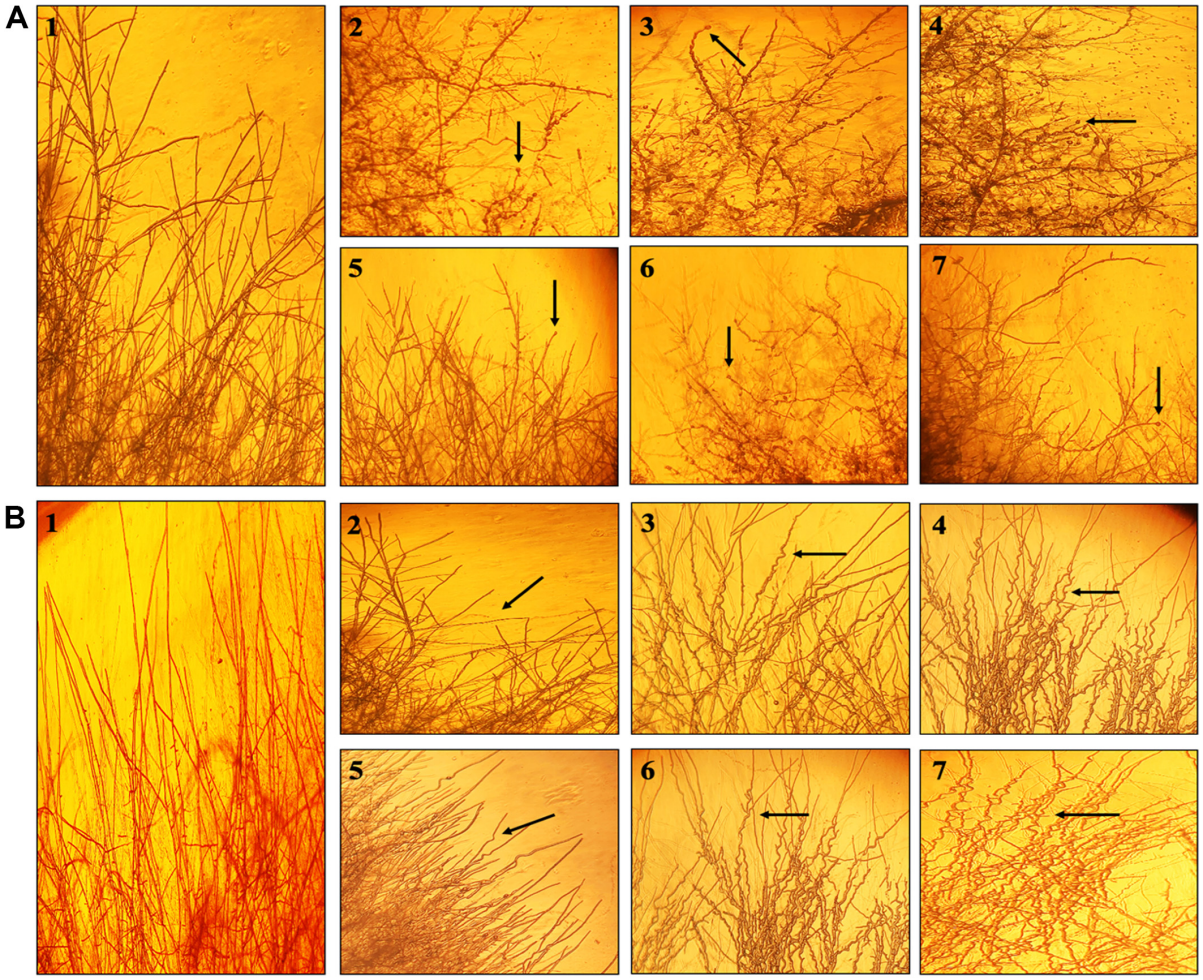
Stereomicroscopy (40X) observations revealing antagonistic activities of compounds against *Alternaria alternata* 1 (A) and *Fusarium equiseti* (B). Elongated hyphae of *Alternaria alternata* 1, rosette formation and hyphal deformation by growing column fractions (**A**1). PB-St2 crude extract (**A**2), PCA (**A**3), PC3 (**A**4), mupirocin (**A**5), 2-OH-phz (**A**6), pyoluteorin (**A**7) with *A. alternata* 1. Elongated hyphal morphology of *Fusarium equiseti* (**B**1), branching of *Fusarium* hyphae by growing with mupirocin (**B**2). Deformed spiral shaped morphology of *Fusarium* with PCA (**B**3), PC3 (**B**4), pyoluteorin (**B**5), 2-OH-phz (**B**6), and PB-St2 crude extract (**B**7).

**Fig. 7:**
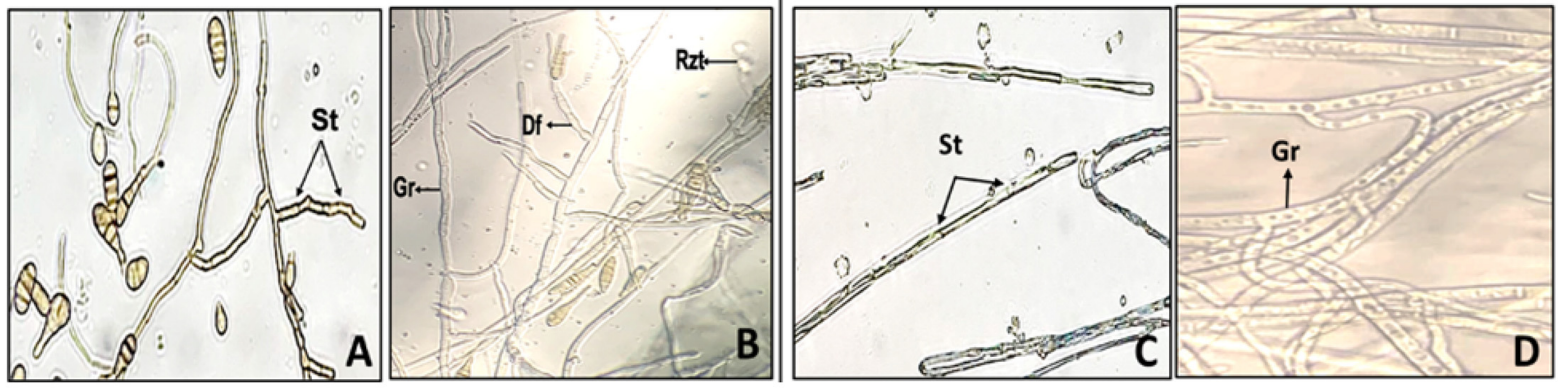
Fluorescent microscopy (254 nm, 40X) observation revealing the effect of PC3 compound on *A. alternata* 1 and F. equiseti. *A. alternata* 1 with septate in hyphae and high sporulation (**A**) PC3 treated *A. alternata* 1 showed granulations in hyphae, rosette formation and deformations (**B**) normal septate hyphae of *Fusarium* (**C**) effect of PC3 compound on hyphae of *Fusarium* (**D**). St: septate, Rzt: rosette formation, Df: deformations, Gr: granulations

Plate assays revealed significant reduction in fungal growth. Percentage inhibition against *Alternaria alternata* 1 ranged from 27% to 89%, where PC3 (pyoluteorin, PCA, 2-OH-phz) and PC2 (PCA) showed the highest inhibition (83% and 89%, respectively) followed by P3 (2-OH-phz; 42.4%), and P1 (pyoluteorin; 37.3%). Against *Alternaria alternata* 2, the highest inhibition activity was observed by PC3 (pyoluteorin, PCA, 2-OH-phz; 73.4%) followed by P3 (2-OH-phz; 62.6%). Similarly, against *Fusarium equiseti*, PC3 (pyoluteorin, PCA, 2-OH-phz) showed the highest antagonistic activity with 39.15% followed by PC2 (PCA; 37.02%), whereas, against *F. incarnatum*, the highest inhibition activity was recorded by PC3 (pyoluteorin, PCA, 2-OH-phz; 52.5%) followed by P3 (2-OH-phz) with 41.5%. Against *Colletotrichum falcatum*, the highest percentage inhibition was observed by PC3 (pyoluteorin, PCA, 2-OH-phz; 58.1%) followed by PC2 (PCA; 49.9%). Mupirocin (PC1) showed lowest percentage inhibition against all the fungal pathogens (Fig. 5). Mycelia of *A. alternata* 1 and 2 treated with PBSt2 compounds, showed rosette formation when observed under stereomicroscope, whereas, against *Fusarium* sp. and *C. falcatum*, hyphae deformed into spiral shaped hyphae (Fig. 6). On the other hand, fluorescent microscopy revealed that spores of *Alternaria* sp. reduced in the treated areas along with deformations, granulations, and condensation in the hyphae. PC2 (PCA) treated *Fusarium* sp. and *C. falcatum* showed swelling and excessive granulations in the hyphae compared to the control (Fig. 7).

### Antibacterial Activity

**Fig. 8:**
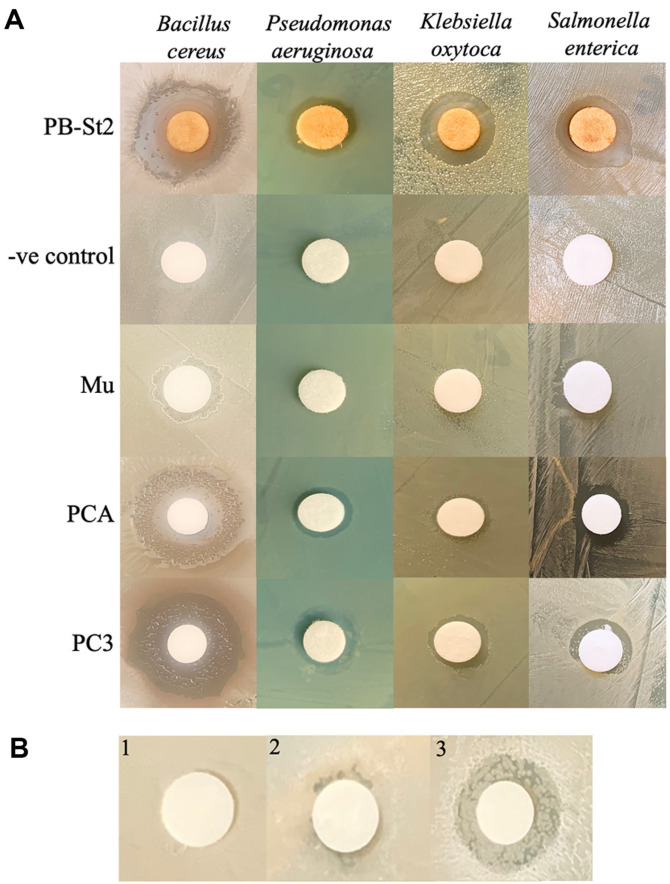
Antagonistic activity of purified compounds against *B. cereus*, *P. aeruginosa*, *K. oxytoca* and *S. enterica* (A). Antagonistic activity of HPLC fractions against *B. cereus*; control (1), pyoluteorin (2), 2-OH-phz (3) (B).

Column collected fractions containing compounds PC1 (mupirocin), PC2 (PCA), and PC3 (pyoluteorin, PCA, 2-OH-phz) were screened for their antagonistic potential against the bacterial pathogens. Positive (PB-St2 crude extract) and negative control (200 μl DMSO + 800 μl methanol) were also tested. Against *Bacillus cereus*, inhibition zones of the isolated compounds PC3 (pyoluteorin, PCA, 2-OH-phz) and PC2 (PCA) increased 1.125 and 1.0625 times as compared to the positive control. The maximum inhibition zone (diameter) was measured for PC3 (pyoluteorin, PCA, 2-OH-phz; 1.8 cm) followed by PC2 (PCA; 1.7 cm), surpassing the zone of wild type crude extract PB-St2 (1.6 cm). Similarly, against *P. aeruginosa*, the antagonistic effect of PC3 (pyoluteorin, PCA, 2-OH-phz) and PC2 (PCA) increased by 1.28 and 1.11 fold when compared to the PB-St2 crude extract. Inhibition zone for PC3 (pyoluteorin, PCA, 2-OH-phz; 0.9 cm) was followed by PC2 (PCA; 0.78 cm) and PB-St2 (0.7 cm). Contrary to this, antibacterial potential of PB-St2 crude extract against *Klebsiella oxytoca* was 1.625 times higher than PCA followed by PC3 (pyoluteorin, PCA, 2-OH-phz; 1.44 times). The biggest inhibitory zone was measured for PB-St2 (1.3 cm) followed by PC3 (pyoluteorin, PCA, 2-OH-phz; 0.9 cm) and PC2 (PCA; 0.8 cm). A similar pattern was observed against *Salmonella enterica* where zone for PB-St2 was 1.09 times bigger than PC3 (pyoluteorin, PCA, 2-OH-phz). The diameter for crude extract of PB-St2 was measured as 1.2 cm followed by PC3 (pyoluteorin, PCA, 2-OH-phz;1.1 cm) and PC2 (PCA;1 cm) (Fig. 8A). As PC3 showed the highest inhibitory activity against *B. cereus*, therefore, HPLC collected compounds (pyoluteorin and 2-OH-phz) were tested only against this organism, at 100 μg. Compound P3 (2-OH-phz) showed antagonistic activity with inhibition zone of 1.2 cm against *B. cereus*. Compound P1 (pyoluteorin) showed weak activity compared to the negative control (Fig. 8B).

### Anticancer Activity

**Fig. 9:**
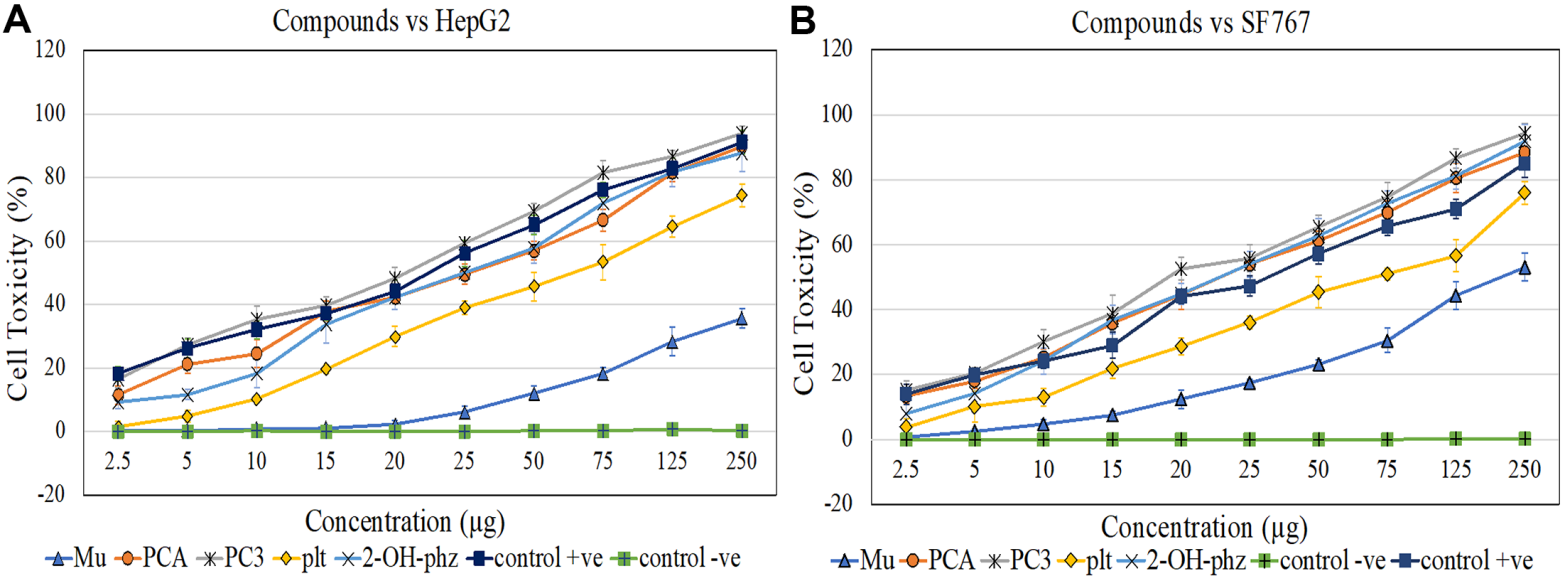
Measurement of IC_50_ of PB-St2 compounds against HepG-2 (A) and, SF767 (B). The error bars represent standard deviation and results are the means of two replicates.

The cytotoxic activity of all fractions was tested against HepG-2 and SF767 cell lines at various concentrations of purified compounds. Positive (PB-St2 crude extract) and negative (200 μl DMSO + 800 μl methanol) controls were also tested. Negative control did not show any effect on either of the cell lines. In case of HepG-2, positive control and PC3 (pyoluteorin, PCA, 2-OH-phz) achieved the highest IC_50_ at a concentration of 25 μg each. However, IC_50_ of PC2 (PCA) and P3 (2-OH-phz) was observed at 50 μg each (Fig. 9A). Alternatively, for SF767 cells PC3 (pyoluteorin, PCA, 2-OH-phz), PC2 (PCA) and P3 (2-OH-phz) achieved their IC_50_ faster than the positive control. PC3 (pyoluteorin, PCA, 2-OH-phz) possessed the highest cytotoxic activity at 20 μg concentration followed by PC2 (PCA) and P3 (2-OH-phz) at 25 μg each. Pyoluteorin (P1) showed its IC_50_ at 75 μg against both the cell lines. Mupirocin (PC1) did not show any significant cytotoxic activity against both cell lines (Fig. 9B). Light microscopy of MTT (3-[4,5-dimethylthiazol-2-yl]-2,5 diphenyl tetrazolium bromide) revealed that the cells grown with the collected fractions reduced the cell numbers with deformed morphology (Fig. S9).

## Discussion

Fluorescent pseudomonads have been widely reported for their plant growth promoting traits and suppression of phytopathogens. The genus comprises several important species among which *Pseudomonas chlororaphis* subsp. *aurantiaca* has gained special attention because of the production of plethora of secondary metabolites including phenazines, hydroxamate-type siderophores, cyclic-lipopeptides, pyrrolnitrin, diketopiperazines, rhamnolipids, and volatiles including hydrogen cyanide [23,24]. These secondary metabolites have been majorly analyzed for their antifungal activities against phytopathogens and their agricultural uses, and only a few of them have been reported for their antibacterial and anticancer properties. To address this gap, this study focused on the characterization of secondary metabolites of *P. chlororaphis* subsp. *aurantiaca* PB-St2 for their anticancer and antibacterial activities in addition to the antifungal potential.

The most abundant metabolites of *P. aurantiaca* are phenazines which comprise diverse strain-specific derivatives. Phenazine derivatives including phenazine-1-carboxylic acid (PCA), 2-hydroxyphenazine (2-OH-phz), 2-hydroxyphenazine-1-carboxylic acid (2-OH-phz-1-COOH), 6-methyl phenazine-1-carboxylic acid (6-methyl-phz-1-COOH), and phenazine 1,6-dicarboxylic acid (phz-1,6-di-COOH) have been previously reported from *P. aurantiaca* PB-St2 [13]. Similarly, phenazine-1-carboxamide (PCN), PCA, 2-OH-phz have been reported from *P. aurantiaca* strains JD37, ST-TJ4, and SWRIQ11 [24, 25]. Nonetheless, there is hardly any report on the optimum temperature and incubation time period for the maximum production of phenazines. When cultivated on different temperatures, *P. aurantiaca* PB-St2 showed the maximum yields of phenazines, dominantly PCA, at 32°C, whereas, the lowest yields of phenazines were observed at 40°C. A similar study for *Pseudomonas* sp. strain M18 previously showed the variability in PCA production at different temperatures, *i.e.* at 28°C, the PCA production was 127 mg/l, which reduced 4 folds at 37°C [26]. Incubation time period showed the optimum production of PCA after 48 h of incubation (587.874 ppm) followed by 72 h of incubation with 545.7755 ppm. Previous studies have also documented the 72 h incubation period as an optimal time for metabolites production and accumulation in the culture medium validating the results obtained in this study [27]. For instance, a previous research conducted by Chandran *et al*. (2014) compared the metabolites production by four bacteria including *B. subtilis*, *Enterococcus hirae*, *Acinetobactor mufti*, and *P. aeruginosa*. *P. aeruginosa* demonstrated the highest pigments and metabolites production after 72 h of incubation, validating the results obtained in this study [28, 29].

Initially, optimization was done through OFAT (One Factor at a Time) approach to analyze the most suitable cultivation period and temperature for phenazine production. On the other hand, to determine the correlation of temperature and incubation period on phenazine production RSM based Box Behnken Design (BBD) was adopted due to its high efficiency with few experiments. RSM is a statistical technique which is useful to design experiments, analyze interaction between independent variables and check response patterns [30]. Both approaches did not contradict massively in their results. According to RSM, the maximum PCA production was at 32°C. Additionally, 72 h of incubation resulted in high phenazine production inferring that results from both OFAT and RSM-BBD approaches projected the same findings. The results determine that temperature and incubation period variability impose a high impact on PCA production.

Previous experiments using PB-St2 indicated that most of the metabolites produced are highly polar in nature. This attribute makes it difficult to select the suitable mobile phase for isolation and purification of compounds. Thin layer chromatography (TLC) is a simple, quick and inexpensive procedure that gives a quick answer as to how many compounds are in the sample. TLC is also used to support the identity of a compound in a mixture when the *Rf* of unknown compound is compared with the *Rf* of a known compound. This has also been used for the confirmation of purity and identity of isolated compounds [31]. Chloroform, acetonitrile, ethyl acetate, and dichloromethane are enlisted among the moderately polar solvents. For this study, the most promising solvent system for TLC was the combination of *n*-hexane and ethyl acetate. Selection of solvents as mobile phase for gravitational column chromatography was a combination of polar (ethyl acetate) and non-polar solvent (*n*-hexane). Gradient solvent system (non-polar to high polar solvent system) is recommended for best elution and best separation of various organic compounds [32, 33]. Based on this solvent system, PB-St2 crude extract yielded three fractions including a greasy textured compound (mupirocin), yellow-colored crystals of PCA, and orange-red colored crystals collectively comprising pyoluteorin, PCA and 2-OH-phz. HPLC detection of phenazines from crude extract of PB-St2 revealed close affinity of PCA and 2-OH-phz indicating their co-elution and orange-red colored crystals in the third fraction. In comparison to other phenazine derivates produced by *P. aurantiaca* PB-St2, 2-OH-phz and PCA are the most vital and abundantly produced phenazines of this strain majorly determining the bioactivities and an overall potential of this strain.

Several bacterial secondary metabolites have been largely reported for their antibacterial, anti-malarial, anthelmintic, antifungal, antiviral, and antitumor compounds. Antifungal properties of *Pseudomonas* spp. metabolites are well documented from agricultural perspective, however, less is explored of their clinical use as antibacterial and anticancer agents, particularly with respect to *P. aurantiaca* metabolites. Compounds of *P. aurantiaca* PB-St2 identified in this study demonstrated considerable antibacterial activities against several human pathogens. A previous study showed inhibitory activity of crude extract of *Pseudomonas* strain DO5 against *S. aureus*, *Enterococcus faecalis*, *Escherichia coli*, *Streptococcus pyogenes*, *K. pneumoniae*, *Proteus mirabilis*, *Vibrio cholera*, and *Salmonella typhi*. The extract also showed antifungal activity against *Candida albicans* [34]. However, the antibacterial and antifungal activities of purified compounds were not shown in that study. In another research, rhamnolipids isolated from *P. aeruginosa* DS9 inhibited several fungal pathogens indicating their use as fungicidal compounds [35]. Similarly, *in vitro* antimicrobial activities of synthetic phenazine derivatives were assessed against methicillin resistant *S. aureus* (MRSA), and *E. coli*. However, their antibacterial effect against *S. aureus* was insignificant as compared to the antibacterial activities of PCA and 2-OH-phz, used in this study [36]. Another study unraveled the iron-chelating and antibacterial activity of *n*-oxide hydroxy-phenazine and its structural analogs against *S. aureus* [37]. Likewise, 6-hydroxyphenazine-1-carboxamide, and methyl 6-carbamoylphenazine-1-carboxylate isolated from soil-derived *Streptomyces* demonstrated antibacterial activity against *Staphylococcus albus*, and *Bacillus subtilis* [38]. Mupirocin, PCA, and 2-OH-phz assessed in this study for antibacterial potential against Gram positive and Gram-negative bacteria showed their superiority being the naturally produced metabolites of *P. aurantiaca*.

In addition to antibacterial activity, *P. aurantiaca* PB-St2 metabolites also demonstrated significant antifungal activities against phytopathogens including *Alternaria* sp., *Fusarium* sp. and *C. falcatum* showcasing it as a promising biofungicide candidate. Inhibition of agricultural pathogens is a well-documented feature of *Pseudomonas* sp. For instance, *P. monteilii* MTCC 9796 was reported to be active against *Colletotrichum falcatum* and *Fusarium moniliforme* [39]. The antifungal effect of *Pseudomonas* sp. AF7 was reported against three plant pathogens including *A. niger*, *A. flavus*, and *F. oxysporum* [40]. Similarly, phenazines from *Pseudomonas fluorescens* 9 exhibited a considerable inhibition of soybean pathogen *Macrophomina phaseolina* [41]. Phenazine1-carboxamide and PCA from *P. aeruginosa* VSMKU1 strain antagonized *Rhizoctonia solani*, *Macrophomina phaseolina*, *Fusarium oxysporum*, *Alternaria alternata* and *Sclerotium rolfsii* causing mycelial arrest in plate assays [42]. Previously, *P. aurantiaca* strain PB-St2 was reported for biocontrol of *Aspergillus* sp. however, the potential of pure compounds was not tested. This study demonstrates the efficacy of phenazines in particular for inhibition of fungal pathogens and shows the efficacy of naturally synthesized phenazines over the chemically synthesized derivatives. Previously, it has been shown that chemical synthesis of phenazines yields lower amounts and the excreted by-products of the reaction including lead oxide, aniline, o-phenylenediamine, and azobenzoate are toxic [43]. Hence, *in vivo* biocatalytic synthesis of phenazines by *P. aurantiaca* strains and the biosafety of *Pseudomonas* sp. as biofungicides and biofertilizers provides an ecofriendly substitute of chemical synthesis and an option for sustainable agricultural practices.

Pharmacological activities of phenazines have been only recently explored. Radical scavenging, topoisomerase inhibition, and polynucleotide interactions are attributed as the potential mechanism for anticancer activity of halogenated aromatic phenazine compounds. Because of the aromatic nature, phenazines intercalate and intervene DNA replication and induce G cell cycle arrest and apoptosis, and inhibit the cell viability of cancerous cells [44]. Recently, synthetic phenazine-1-carboxylic acylhydrazone derivatives were assessed for anticancer activities against HeLa and A549 cancer cell lines with significant IC_50_ values as compared to in-practice chemotherapeutic drugs [45]. A 2-OH-phz resembling compound n-(2-hydroxyphenyl)-2-phenazinamine from *Nocardiopsis exhalans* was reported for induction of p53-mediated intrinsic apoptosis signaling in lung cancer cell lines [46]. Nevertheless, the more focus of the anticancer activities of phenazines is towards synthetic compounds. *P. aurantiaca* PB-St2 phenazine compounds including PCA and 2-OH-phz used in this study showed considerable 1C50 values of 25 μg against HepG-2 cancer cell line. The mixture of these two phenazines showed 1C50 value at 20 μg which was significantly higher than the positive control and synthetic phenazine derivative reported in other studies [47]. The anticancer potential of phenazines of *P. aurantiaca* demonstrates the manifold attributes possessed by this genus.

Conclusively, metabolites such as phenazines can be highly valuable in agricultural, pharmacological, and industrial sectors. Evaluation of *P. aurantiaca* PB-St2 metabolites for their antibacterial and anticancer potential demonstrate their use beyond agricultural applications. Higher anticancer activities of phenazine-1-carboxylic acid (PCA), 2-hydroxyphenazine (2-OH-phz) and pyoluteorin than the synthetic phenazine derivatives indicate their superiority, cost-effectiveness, and eco-friendliness. The optimization of temperature and growth parameters can be further assessed for industrial production of these compounds for multifaceted applications in agriculture and pharmacological sectors.

## Supplemental Materials

Supplementary data for this paper are available on-line only at http://jmb.or.kr.



## Figures and Tables

**Table 1:** List of bacterial and fungal strains used in this study.

Strains	Isolation Source	Accession No.
Bacterial Pathogens		
*Bacillus cereus*	Lettuce leaves	MZ414206
*Pseudomonas aeruginosa*	Clinical isolate	OP740518
*Klebsiella oxytoca*	Clinical isolate	MZ414202
*Salmonella enterica*	Poultry meat	MZ414205
Fungal Pathogens		
*Alternaria alternata* 1 (green)	Wood of Lemon *Eucalyptus*	OP740511
*Alternaria alternata* 2 (black)	Wood of Lemon *Eucalyptus*	OP740510
*Fusarium equiseti*	Chickpea	MN636869
*Fusarium incarnatum*	Rice	MN636870
*Colletotrichum falcatum*	Sugarcane	-

**Table 2:** Gradient run used for HPLC analysis of crude extract.

TIME (minutes)	Water % (Solvent A)	Acetonitrile % (Solvent B)
0.0	95	5
1.0	95	5
15.0	5	95
30.0	5	95

**Table 3:** Independent variable factors and the levels for designing the Kindly use the term Box Behnken Design (BBD).

Factor	Name	Units	Low	High
A	Temperature	°C	28	40
B	Incubation period	Hours	24	96
