# Supplementary material for: Assessment of Anticancer and Antimicrobial Potential of Bioactive Metabolites and Optimization of Culture Conditions of Pseudomonas aurantiaca PB-St2 for High Yields
Source: J Microbiol Biotechnol. 2025 Feb 14;35:e2311041. doi: 10.4014/jmb.2311.11041 (PMC11883349; doi:10.4014/jmb.2311.11041)
Supplement: Supplementary file 1 [file jmb-35-e2311041-supple.pdf]

J. Microbiol. Biotechnol. 2025. 35: e2311041
https://doi.org/10.4014/jmb.2311.11041

Supplementary Table and Figures

Assessment of Anticancer and Antimicrobial Potential of Bioactive Metabolites and Optimization of Culture Conditions of *Pseudomonas aurantiaca* PB-St2 for High Yields

Table S1. BBD for two variables with experimental design and responses (extract weight, PCA quantification and optical density).

Run	Factor 1<br>A: Temperature (°C)	Factor 2<br>B: Incubation Period (Hours)	Response 1<br>Extract weight (mg)	Response 2<br>PCA Quantification (ppm)	Response 3<br>Optical Density (600 nm)
**1**	34	24	10.99±2.23	139.14±1.11	0.87±0.06
**2**	32	96	67.65±1.76	545.775±1.98	4.06±0.13
**3**	28	60	29.3±1.22	302.66±0.86	2.16±0.28
**4**	40	24	5.01±0.88	85.3±0.05	0.42±1.09
**5**	32	60	59.11±2.32	518.93±1.32	3.31±0.85
**6**	40	48	5.5±0.05	93.99±0.07	0.67±0.03
**7**	28	96	35.25±2.65	378.88±2.14	2.71±0.66
**8**	40	96	11.1±0.07	99.987±2.24	0.677±0.07
**9**	30	96	65.3±2.09	515.675±1.16	4.99±0.44
**10**	32	60	60.21±2.72	533.77±1.98	3.81±0.85
**11**	30	60	57.65±1.11	477.211±1.01	3.34±0.21
**12**	34	72	41.65±1.73	259.45±0.99	3.14±0.76
**13**	28	24	15.22±1.88	232.8±0.45	1.28±1.23

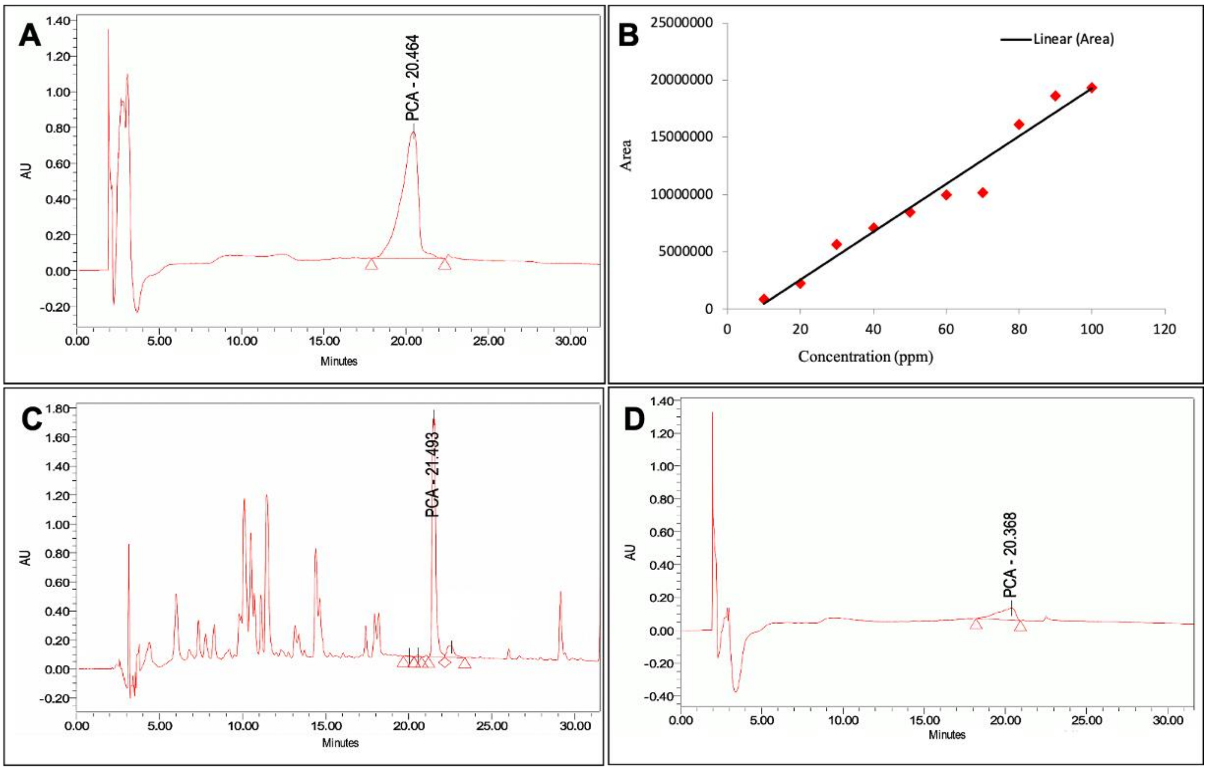

Fig. S1. HPLC chromatograms for quantification of PCA (**A**) Standard run of PCA. (**B**) Standard curve of PCA standard. (**C**) HPLC chromatogram showing maximum production of PCA at 32°C. (**D**) HPLC chromatogram showing lowest production of PCA at 40°C.

A. PCA Quantification

Source	Sum of Squares	df	Mean Square	F-value	p-value	
Model	335.97	5	67.19	9.77	0.0046	significant
A-Temperature	84.82	1	84.82	12.33	0.0098	
B-Incubation period	0.0023	1	0.0023	0.0003	0.9860	
AB	0.8114	1	0.8114	0.1180	0.7413	
A²	116.12	1	116.12	16.88	0.0045	
B²	17.98	1	17.98	2.61	0.1499	
Residual	48.15	7	6.88			
Lack of Fit	48.15	4	12.04			
Pure Error	0.0000	3	0.0000			
Cor Total	384.13	12				

B. Extract weight

Source	Sum of Squares	df	Mean Square	F-value	p-value	
Model	68.40	5	13.68	14.78	0.0013	significant
A-Temperature	8.12	1	8.12	8.77	0.0211	
B-Incubation period	8.50	1	8.50	9.18	0.0191	
AB	0.0365	1	0.0365	0.0394	0.8483	
A²	15.12	1	15.12	16.33	0.0049	
B²	7.56	1	7.56	8.16	0.0244	
Residual	6.48	7	0.9259			
Lack of Fit	6.48	4	1.62			
Pure Error	0.0000	3	0.0000			
Cor Total	74.88	12				

C. Optical Density

Source	Sum of Squares	df	Mean Square	F-value	p-value	
Model	3.55	5	0.7107	24.81	0.0003	significant
A-Temperature	1.12	1	1.12	39.19	0.0004	
B-Incubation period	0.9534	1	0.9534	33.28	0.0007	
AB	0.1493	1	0.1493	5.21	0.0564	
A²	0.2207	1	0.2207	7.70	0.0275	
B²	0.0742	1	0.0742	2.59	0.1516	
Residual	0.2005	7	0.0286			
Lack of Fit	0.2005	4	0.0501			
Pure Error	0.0000	3	0.0000			
Cor Total	3.75	12				

Fig. S2. Experimental data obtained for the responses based on RSM (A) PCA quantification, (B) Extract weight and (C) Optical density.

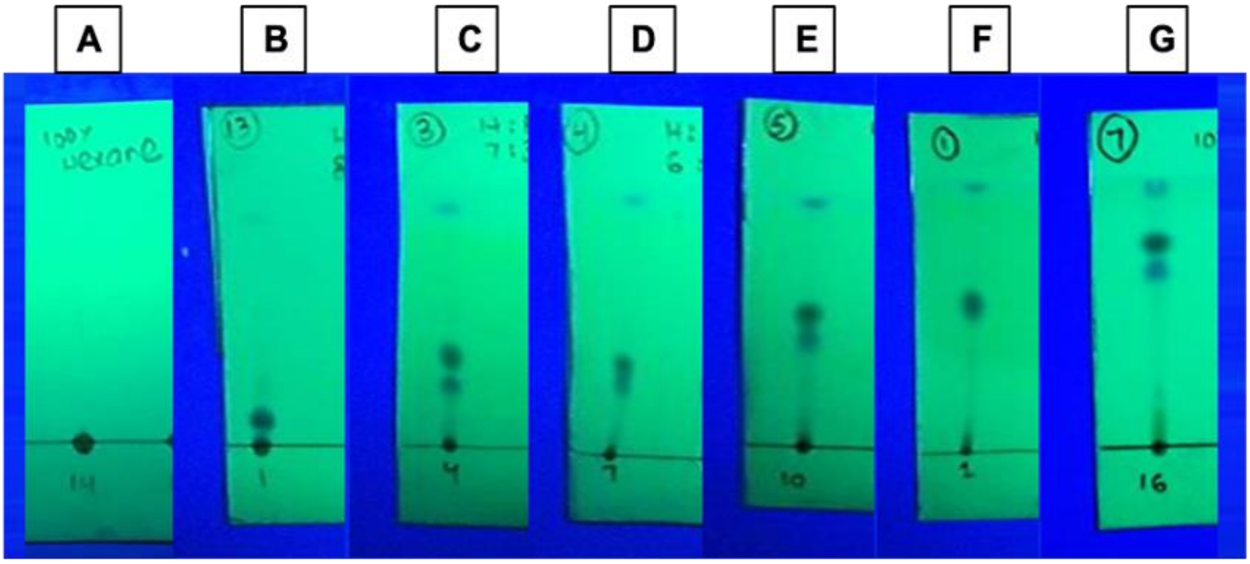

Fig. S3. Gradient of n-Hexane and ethyl acetate for methanol extract. a to g: (1:0, 8:2, 7:3, 6:4, 1:1, 4:6, 0:1).

Table S2. Detailed analysis of compounds eluted during column chromatography.

Mobile Phase	Ratio	Combined Fractions	Amount of Mobile Phase (ml)	Rf value	Yield (mg)
n-Hexane	100	-	50	-	-
n-Hexane: Ethyl acetate	9:1	-	50	-	-
	8:2	-	50	-	-
	7:3	F1-F8 (PC1)	100	1	19.7
	6:4	-	50	-	-
	1:1	F15-F28 (PC2)	300	0.36	70.35
	4.7:5.3	-	50	-	-
	4.6:5.4	-	50	-	-
	4.5:5.5	F31-F48 (PC3)	300	0.26	120
	4.4:5.6	-	50	-	-
	4.1:5.9	-	50	-	-
	3.8:6.2	-	50	-	-
	3.5:6.5	-	50	-	-
	3.2:6.8	-	50	-	-
	2:8	-	50	-	-
	1:9	-	50	-	-
Ethyl acetate	100	-	50	-	-
Ethanol	100	-	50	-	-

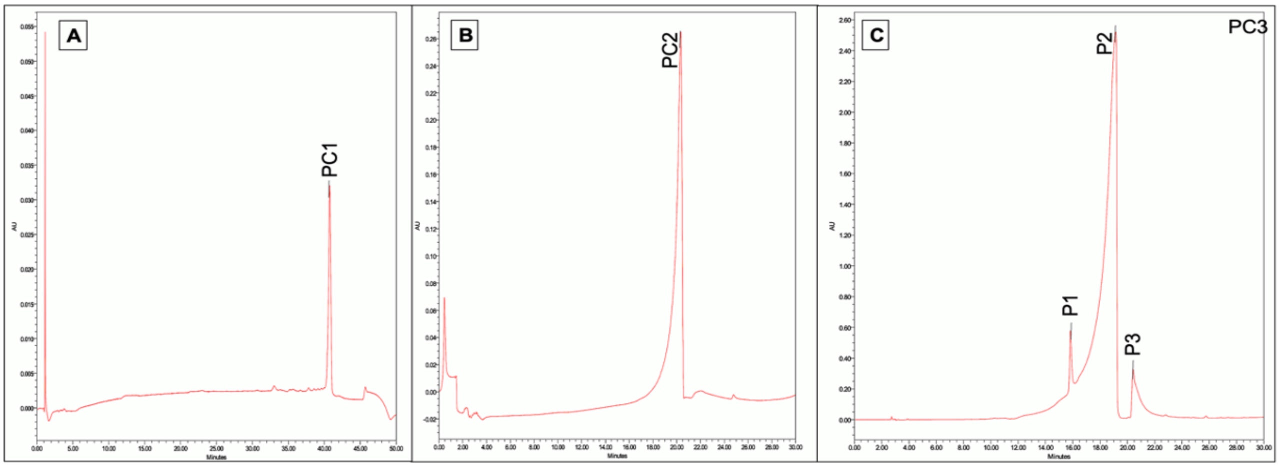

Fig. S4. HPLC Chromatogram of column collected fractions (A) PC1, (B) PC2 revealing single peaks and (C) PC3 compound revealing three peaks (P1, P2, P3).

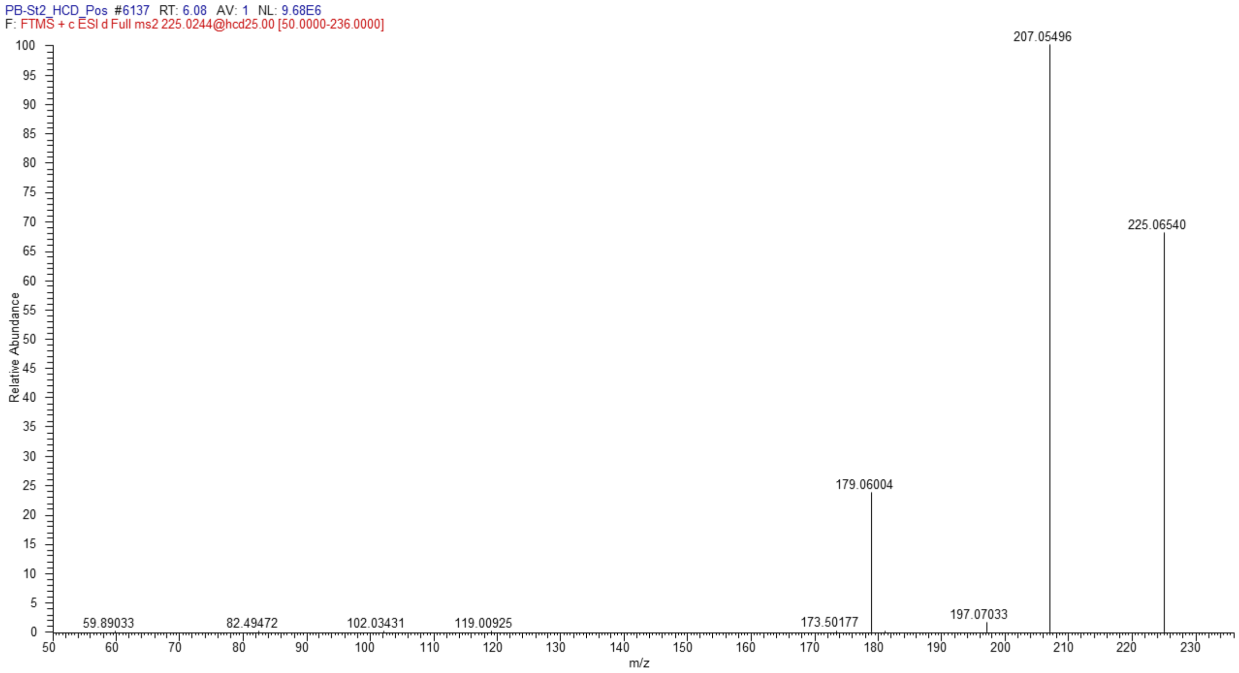

Fig. S5. MS/MS mass spectrum representing PCA m/z $[M+H]^+$ 225.0658 ion specie in positive ion mode analysis.

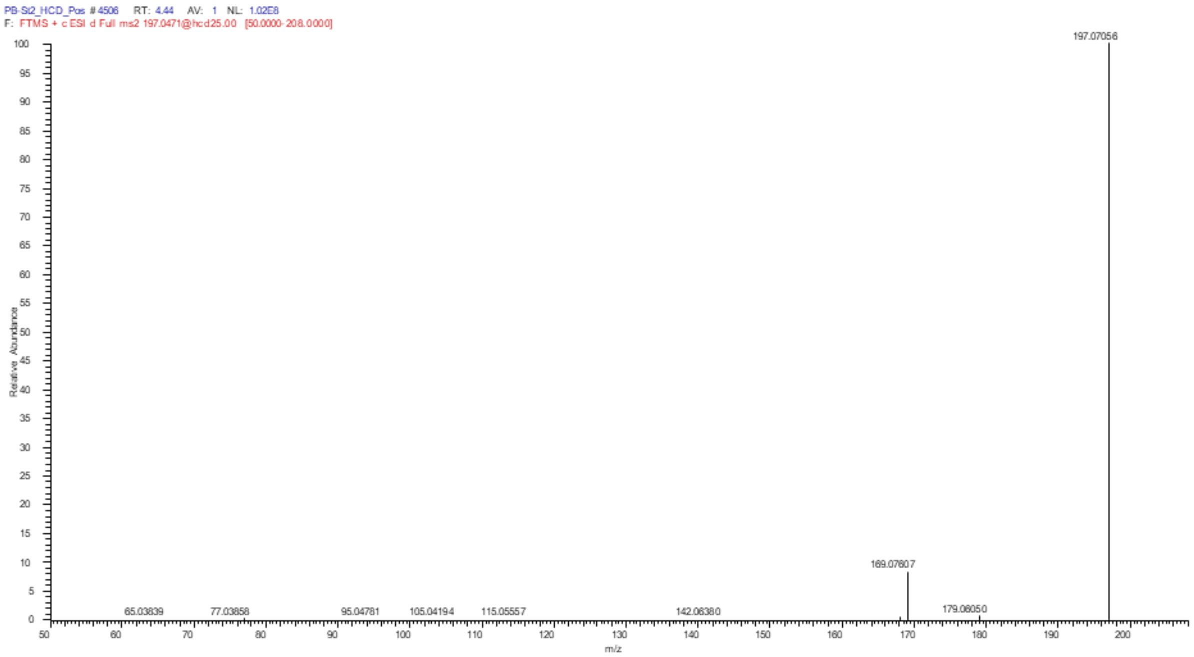

Fig. S6. MS/MS mass spectrum representing 2-OH-Phz m/z $[M+H]^+$ 197.0703 ion specie in positive ion mode analysis.

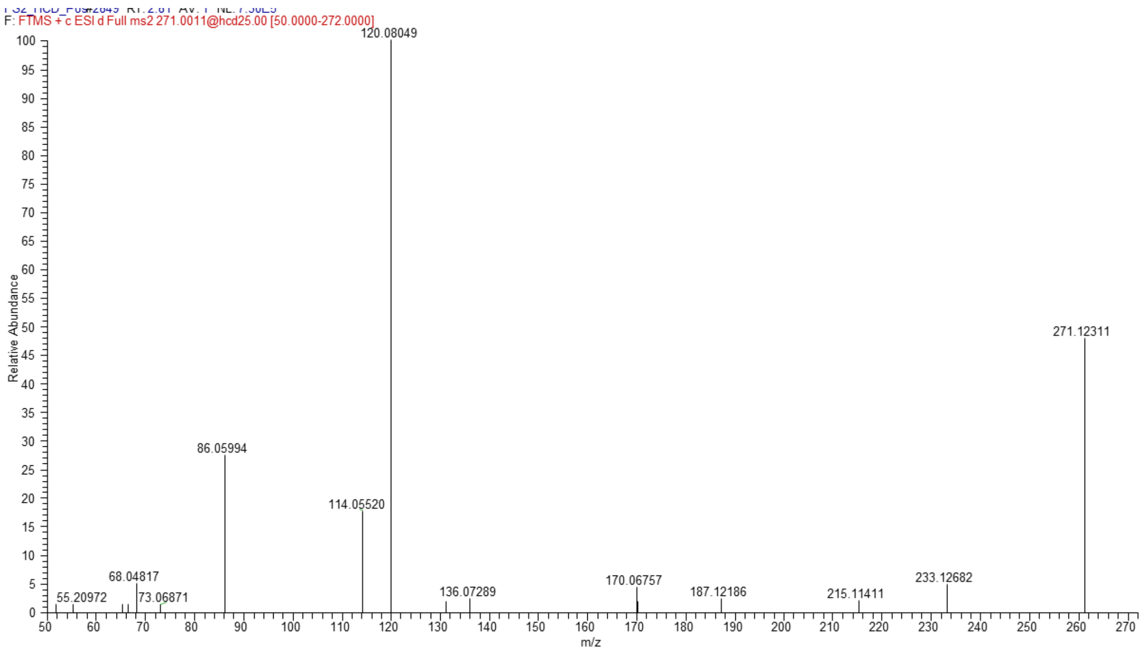

Fig. S7. MS/MS mass spectrum representing pyoluteorin m/z $[M+H]^+$ 271.1 ion specie in positive ion mode analysis.

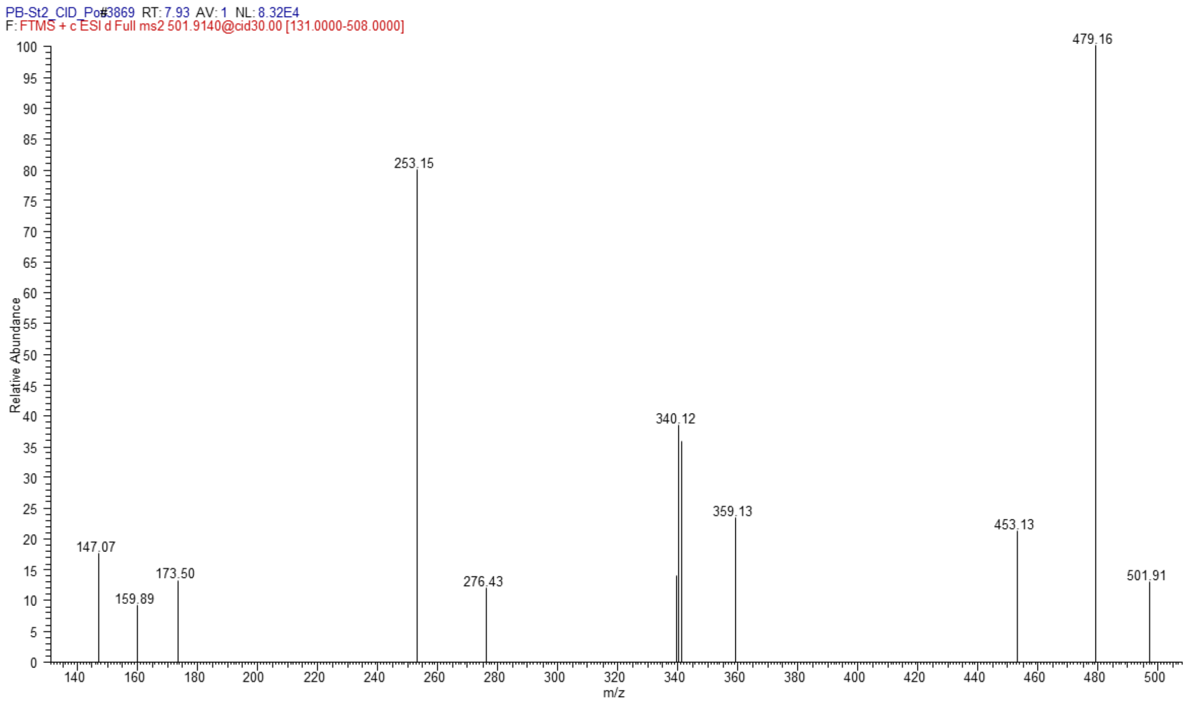

Fig. S8. MS/MS mass spectrum representing mupirocin m/z $[M+H]^+$ 501.91 ion specie in positive ion mode analysis.

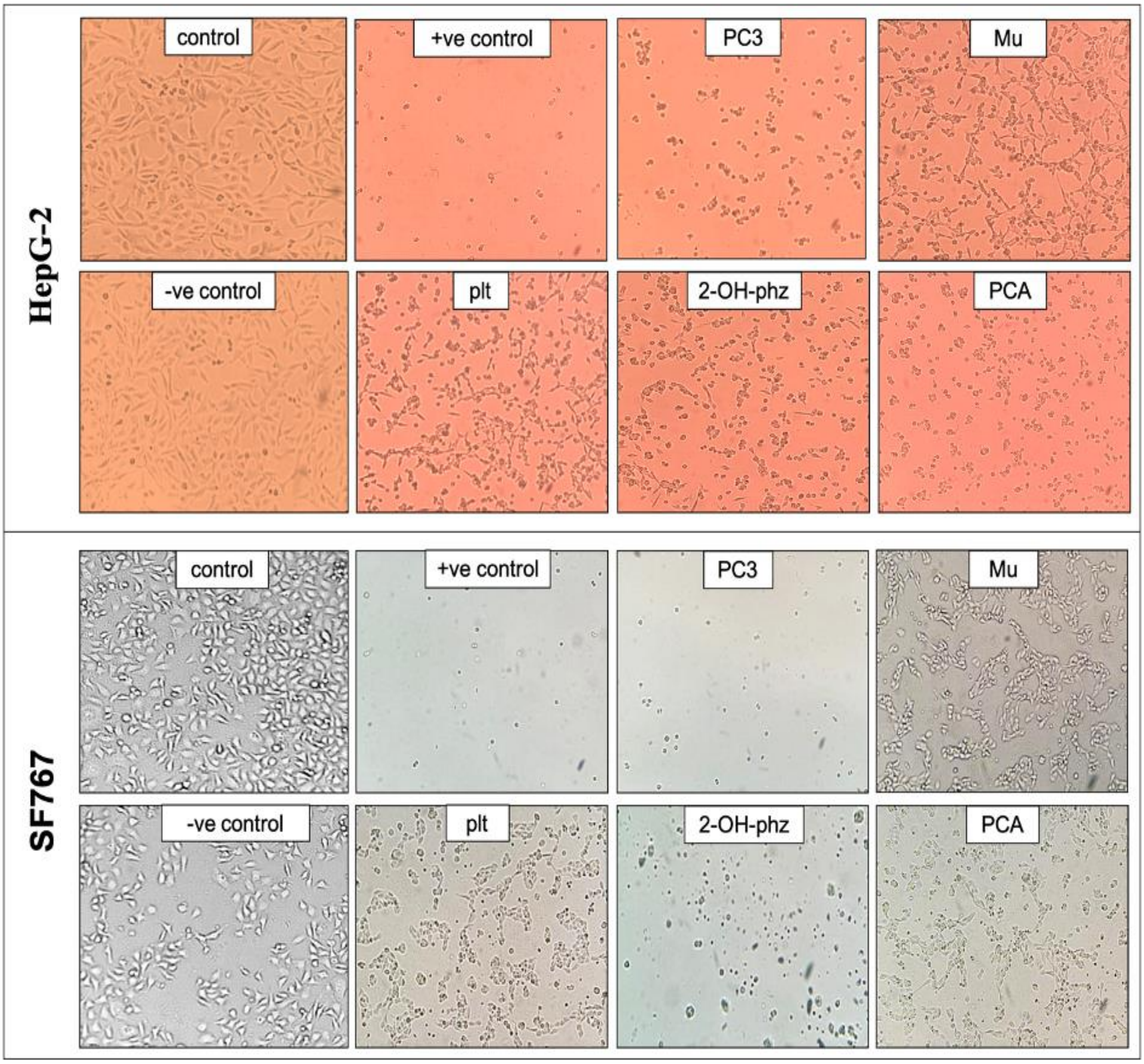

Fig. S9. Light microscopy (40X) observation revealing the effect of column collected compounds [Mupirocin (Mu), PCA, PC3] and HPLC collected compounds [Pyoluteorin (Plt), 2-Oh-phz] on HepG-2 and SF767 cell lines. Control and negative (-ve) control depict morphology of healthy cells. PB-St2 crude extract was used as positive control (+ve control).
